# Editorial: Clinical trials in drug metabolism and transport: 2025

**DOI:** 10.3389/fphar.2026.1952763

**Published:** 2026-08-27

**Authors:** Yurong Lai, Zhihao Liu, Yili Ding

**Affiliations:** 1 Gilead Sciences Inc, Foster City, CA, United States; 2 Resolian, Malvern, PA, United States; 3 Kean University-Wenzhou, Wenzhou, China

**Keywords:** bioequaivalence, biosimilar, clinical pharmacology, hepatic impairment, human ADME studies, pharmacokinetics and pharmacodynamics (PKPD)

Clinical drug metabolism and transport studies provide the quantitative foundation for understanding how medicines are absorbed, distributed, metabolized, and eliminated, and how these processes influence efficacy, safety, and dose selection. Building on the successful research topic “Clinical Trials in Drug Metabolism and Transport: 2022”, this topic underscored the essential role of clinical pharmacokinetic trials in drug development and highlighted a broad methodological spectrum, including first-in-human studies, bioequivalence, population pharmacokinetic (PK) analyses, physiologically based pharmacokinetic (PBPK) modeling, human mass-balance, and drug–drug (DDI) and drug–food interaction studies. As stated in 2022 editorial, it also emphasized the value of publishing negative or inconclusive findings, which might otherwise remain inaccessible or be difficult to interpret outside regulatory reports and clinical trial registries.

The current Research Topic, “Clinical Trials in Drug Metabolism and Transport: 2025,” comprising 10 research articles, reflects the field’s progress beyond merely describing plasma concentration–time profiles or calculating traditional PK parameters. Modern clinical studies increasingly aim to link exposure data with developmental physiology, organ dysfunction, route of administration, active metabolites, formulation performance, pharmacologic activity, and treatment accessibility. The collection explores topics such as neonatal enzyme development, hepatic impairment, first-in-human dose escalation, topical delivery, radiolabeled mass balance and metabolite identification, generic bioequivalence, biosimilar comparability, and a controlled test of a common behavioral assumption. Collectively, these studies demonstrate a shift from descriptive pharmacokinetics to a focus on decision-oriented clinical pharmacology.

A notable contribution is the study by Wang and colleagues on CYP1A2 ontogeny and caffeine metabolism in preterm neonates. Although caffeine is used almost universally to treat apnea of prematurity, neonatal dosing remains largely weight-based despite substantial developmental and clinical variability. Using plasma samples from neonates receiving caffeine in intensive care, the researchers measured caffeine and paraxanthine levels and used their metabolic ratio as an *in vivo* indicator of CYP1A2 activity. Although CYP1A2-mediated paraxanthine formation is generally expected to be minimal in preterm neonates, the detection of paraxanthine in all samples suggests measurable CYP1A2 activity even at this early developmental stage. Furthermore, the metabolic ratio increased with postmenstrual age; however, distinct longitudinal patterns indicated that illness severity and other clinical factors also influenced enzyme activity. The authors then systematically applied published neonatal PBPK models and found that these models overpredicted caffeine exposure, highlighting the importance of testing and refining models with real-world data from the target populations. Generalized ontogeny functions based on older infants or more stable groups might not accurately represent extremely premature and critically ill neonates. This research highlights both the potential and constraints of model-informed drug development and advocates moving from weight-based dosing to physiology-informed neonatal pharmacotherapy.

Two studies investigate the consequences of hepatic impairment, addressing the clinically important question of when altered liver function produces a meaningful change in drug exposure. Li and colleagues evaluated ritanine, an intravenous fixed-dose combination of fosrolapitant and palonosetron used to prevent chemotherapy-induced nausea and vomiting. In participants with moderate hepatic impairment, fosrolapitant exposure increased by approximately 50%, but exposure to its active parent drug, rolapitant, was generally comparable to that in healthy controls. Palonosetron exposure was numerically increased, but the totality of PK and safety data did not indicate a clinically meaningful effect that requires dose adjustment. Although the small sample size and exclusion of patients with severe hepatic impairment limit generalizability, the study provides a rational basis for using ritanine without routine adjustment in moderate hepatic dysfunction. On the other hand, Mai and colleagues evaluated SHR0302, a selective Janus kinase (JAK) 1 inhibitor, in subjects with mild or moderate hepatic impairment. Mild hepatic impairment had minimal effect on parent drug exposure. In moderate impairment, maximum concentration was approximately 17% lower, whereas overall exposure remained essentially unchanged. Exposure to the oxidative metabolite SHR161279 decreased by approximately 21%–38% in the hepatic impairment groups. Because the metabolite contributes relatively little to total pharmacologic activity, the decrease was not considered clinically relevant, and dose adjustment was not recommended for mild or moderate impairment. Together, these two hepatic impairment studies reinforce the need to move beyond simple percentage differences in area under the curve or maximum concentration. Parent drug, prodrug, active and inactive metabolites, unbound exposure, pharmacologic activity, and safety margins must be interpreted as an integrated system.

Early drug development is exemplified by the first-in-human study of XT1061, an oral allosteric modulator of the hepatitis B virus core protein. Li and colleagues conducted a randomized, placebo-controlled, single-ascending-dose phase across doses of 12.5–600 mg, a food-effect assessment, and a multiple-dose evaluation at 250 mg. XT1061 was absorbed rapidly, with systemic exposure increasing approximately dose-proportionally and steady state reached within about 2 days, with limited accumulation. A high-fat meal reduced the maximum concentration by about 50% without materially changing total exposure, indicating an effect on absorption rate rather than extent. The compound was generally well tolerated, supporting continued development in patients with chronic hepatitis B. The study exemplifies how first-in-human trials can support more than initial safety characterization. By integrating PK, tolerability, food effect, preclinical potency, and anticipated therapeutic exposure, the investigators generated a rationale for selecting doses for efficacious studies.

Deng and colleagues explored an alternative strategy to improve the therapeutic index by delivering tofacitinib tartrate topically as HZ-J001 cream. Single applications covering 20%–30% of the body surface area produced low systemic concentrations. Repeated twice-daily administration led to substantial accumulation ratios due to slow absorption and prolonged apparent elimination, yet absolute systemic exposure remained low compared with oral tofacitinib. Exposure increased less than proportionally with topical dose, possibly reflecting a limit to percutaneous absorption. This study demonstrates that route of administration can be used to separate local efficacy from systemic toxicity, particularly for drug classes such as JAK inhibitors that have known systemic safety concerns and emphasizes the increasingly important relationship between formulation design and clinical pharmacology.

Liu and colleagues thoroughly examined how JP-1366 (zastaprazan), a potassium-competitive acid blocker, is absorbed, metabolized, and excreted in humans. About 94.3% of the radioactive dose was recovered, with 51.9% in feces and 42.4% in urine. No parent drug was found in the excreta, indicating extensive metabolism. They identified 57 metabolites, mainly formed through oxidation and glucuronidation. The total drug-related radioactivity had a much longer apparent half-life than JP-1366 itself, suggesting the presence of metabolites that are eliminated more slowly. The mass-balance data highlight the roles of renal and fecal elimination, identify circulating and excreted metabolites, and support future evaluations of drug interactions, organ impairment, and metabolite safety. This study reaffirms that human investigations remain indispensable, even with advanced PBPK modeling and computational approaches, because such methods can integrate and extend empirical findings but cannot yet replace foundational human data.

Two studies address generic bioequivalence and the role of clinical pharmacology in improving access to medicine. Yu and colleagues compared a test formulation of vonoprazan fumarate with the reference product Takecab under fasting and fed conditions. The 90% confidence intervals for the geometric mean ratios of maximum concentration and overall exposure fell within the conventional 80%–125% bioequivalence limits in both conditions. Food altered the absorption profile and increased variability but did not change the conclusion of bioequivalence. The study supports therapeutic interchangeability of the two formulations and illustrates the efficiency of the randomized crossover design in controlling between-subject variability. Li and colleagues assessed testosterone undecanoate soft capsules in a four-period crossover study involving healthy postmenopausal women under fed conditions. Studying testosterone undecanoate is complex due to high within-subject variability, natural testosterone levels, and food-dependent lymphatic absorption. After applying baseline correction and reference-scaled average bioequivalence methods, the authors demonstrated that the test and reference products satisfied bioequivalence criteria for peak concentration and overall exposure, while showing comparable safety and tolerability profiles. Although extrapolating results from postmenopausal women to men undergoing testosterone therapy requires caution, the study provides a validated regulatory pathway that could facilitate wider access to the product. This research emphasizes that bioequivalence studies need to be tailored to PK characteristics rather than using a one-size-fits-all approach.

Comparability at the biologic level was examined by Zhou and colleagues in a systematic review and meta-analysis of 7 randomized trials involving 2,829 participants with neovascular age-related macular degeneration who were treated with aflibercept biosimilars or reference aflibercept. No significant differences were observed in best-corrected visual acuity, central subfield thickness, choroidal neovascular lesion outcomes, serious ocular or systemic adverse events, or anti-drug antibodies. A small numerical increase in overall treatment-emergent adverse events in the biosimilar group was not statistically significant. These findings support comparable clinical performance and suggest that biosimilars may reduce the cost of long-term intravitreal anti-vascular endothelial growth factor therapy. Nevertheless, biosimilars cannot be treated simply as small-molecule generics; comparability depends on an integrated evaluation of analytical attributes, biologic activity, pharmacokinetics, efficacy, safety, and immunogenicity. Because each biosimilar was generally represented by a limited number of trials and follow-up was relatively short, continued product-specific pharmacovigilance remains necessary.

The collection also includes a randomized crossover study by Imai and colleagues that tested whether intermittent water consumption during alcohol intake alters ethanol disposition or alleviates hangover symptoms. Thirteen healthy Japanese men with wild-type aldehyde dehydrogenase two consumed a standardized amount of sake either alone or with water. The authors found that water intake did not significantly alter exhaled ethanol or acetaldehyde concentrations, psychomotor performance, or subjective symptoms such as headache, nausea, sleepiness, or impaired concentration. When alcohol dose and drinking rate were controlled, additional water did not accelerate ethanol clearance or prevent hangover. The result does not exclude other benefits of hydration or the possibility that drinking water may reduce total alcohol intake in uncontrolled settings. Instead, it distinguishes a behavioral harm-reduction strategy from a direct PK or PD effect. This negative result directly reflects the previous editorial for the 2022 research topic call, which called for the publication of clinical trials regardless of whether they produce positive outcomes. Such well-controlled negative studies prevent unsupported assumptions from becoming established as clinical advice.

Clinical pharmacology now aims not only to describe post-administration drug behavior but also to explain why exposure varies, assess whether this variation is significant, and determine how this knowledge should inform development or patient care. Across these ten papers, the 2025 collection expands on the 2022 foundation by embracing a more integrated and translational approach to discipline. First, empirical clinical PK data remain essential. Whether the study involves neonatal caffeine, topical tofacitinib, or a generic product, making reliable decisions depends on high-quality human observations. Second, modeling and simulation are most effective when validated against empirical data and adjusted when predictions are inaccurate. Third, variability should be understood through mechanistic analysis. Factors such as developmental age, liver function, skin integrity, food intake, formulation, endogenous baseline concentrations, and metabolite activity influence exposure via different pathways and should not be oversimplified as generic demographic covariates. Furthermore, PK differences are clinically relevant only when considered alongside pharmacodynamics, therapeutic index, safety, and the specific clinical decision. Similarly, a decrease in an inactive metabolite may have little clinical impact, whereas small changes in an active or toxic metabolite could necessitate intervention. Lastly, clinical studies on metabolism and transport directly support equitable access by enabling the development of generics and biosimilars, rational use in patients with organ impairment, pediatric dosing, and locally acting formulations, thereby expanding access to safe and effective therapies.

We sincerely appreciate all authors, reviewers, and editors for their valuable contributions to this Research Topic in Frontiers in Pharmacology.

